# Aberrant Development of Hippocampal GABAergic Neurons Arising from Hypothyroidism Contributes to Memory Deficits in Mice Through Maf Suppressing *Mef2c*

**DOI:** 10.3390/biomedicines13061436

**Published:** 2025-06-11

**Authors:** Mengyan Wu, Xingdong Zeng, Yongle Cai, Haonan Chen, Hao Yang

**Affiliations:** 1 Institute for Fetology, The First Affiliated Hospital of Soochow University, Suzhou 215006, China; 2 School of Basic Medical Sciences, Ningxia Medical University, Yinchuan 750004, China

**Keywords:** developmental hypothyroidism, memory defects, Maf, *Mef2c*, GABAergic interneurons, parvalbumin-positive interneurons

## Abstract

**Background/Objectives:** Thyroid hormone (TH) deficiency during the pregnancy and lactation periods leads to enduring memory impairments in offspring. However, the mechanisms underlying the cognitive and memory deficits induced by developmental hypothyroidism remain largely unexplored. **Methods:** Mice were exposed to propylthiouracil (PTU) or purified water to detect changes in hippocampal neurogenesis and differentiation of their offspring to explain the pathogenesis of impaired learning and memory. In addition, HT22 cell line were used to investigate the regulation between Maf and *Mef2c*. **Results:** Our findings indicate that developmental exposure to PTU results in abnormalities of the preferential differentiation of GABAergic interneurons and a subsequent reduction in PV^+^ inhibitory interneurons in the hippocampus of mouse pups. More significantly, we also indicate that the downregulation of Maf and the consequent alteration of *Mef2c* are likely responsible for the mechanisms through which developmental hypothyroidism influences the differentiation and development of PV^+^ inhibitory interneurons in offspring. **Conclusions:** Consequently, the aberrant development of PV^+^ interneuron in the hippocampus of mice subjected to developmental hypothyroidism potentially contributes to memory deficits during adolescence and adulthood.

## 1. Introduction

Thyroid hormones, specifically L-thyroxine (T4) and 3,5,30-triiodothyronine (T3), play a crucial role in normal brain development. Hypothyroidism during developmental stages in animals can lead to various alterations in behavior, cognition, and memory, among other neurological effects. A multi-center cohort study reported that approximately 7.5% of pregnant women in China suffer from prenatal hypothyroidism [1]. A large amount of evidence further underscores developmental hypothyroidism as a critical risk factor for memory impairments, intellectual disabilities, and autism spectrum disorders [2,3,4]. Notably, THs play a pivotal role in neuronal differentiation, development and processes that are fundamental to memory formation and consolidation [5,6]. It is well-known that the hippocampus has long been thought to be a crucial brain region for cognition and memory, and neuronal degeneration or loss in this area can significantly destabilize neural circuit networks, resulting in hippocampal dysfunction. Within the hippocampus, two major types of neurons—excitatory glutamatergic and inhibitory GABAergic neurons—form the cytoarchitectural and functional basis of neural networks though interareal axon projections [7,8]. Notably, GABAergic local circuit inhibitory interneurons account for approximately 10–15% of the total hippocampal neurons [9]. The excitability of these GABAergic neurons, along with their input and output synaptic transmission, is intricately linked to neuronal differentiation and maturation during late fetal and early postnatal hippocampal development [10,11,12,13]. GABAergic neurons are primarily categorized into three major types of neurons: parvalbumin (PV^+^), somatostatin (SST^+^), and the ionotropic serotonin receptor 5HT3a (5HT3aR^+^) interneurons. Among these, PV^+^ interneurons constitute ~40% of the GABAergic neurons and are vital for memory processes by rapidly encoding information through potent yet transient inhibition of principal excitatory hippocampal neurons. Memory dysfunction is likely attributable to the deficiency and degeneration of PV^+^ neurons [14,15,16]. However, it remains unclear whether developmental hypothyroidism-induced memory impairments in offspring are linked to alterations in PV^+^ interneurons, and the underlying mechanisms remain to be elucidated.

Accumulating evidence indicates that the large Maf transcription factor family, including Mafa, Mafb, c-Maf, and Nrl, has emerged as a pivotal regulator of mammalian gene expression, playing significant roles in metabolic syndrome, osteoarthritic chondrocytes, and cancer [17,18]. Among these, c-Maf, a member of this transcription factor family, regulates cell identity in the nervous system by binding to DNA via its basic leucine zipper motif. In addition, Maf transcription factors play compensatory functions by suppressing the formation of SST^+^ interneurons while promoting PV^+^ interneuron differentiation in secondary progenitors of the medial ganglionic eminence [19,20]. Furthermore, conditional deletion of *Maf* and *Mafb* leads to a reduction in PV^+^ interneurons [19], implying the importance of Maf in the neurogenesis and development of PV+ interneurons. Nevertheless, it remains largely unknown whether the alterations in PV^+^ neurons in developmental hypothyroidism are associated with Maf, and current studies rarely address the involvement of Maf in developmental hypothyroidism. In contrast, *Mef2c* plays a critical role in neuronal differentiation and synapse development, and it is implicated in neurodevelopmental disorders through modulating cortical inhibitory and excitatory synapses [21]. Strikingly, *Mef2c* differential transcription influences interneuron fate, and its deletion disrupts the expression of neurodevelopment-associated genes. Intriguingly, *Mef2c* heterozygosity in developing GABAergic interneurons reduced the intrinsic excitability of PV^+^ interneurons and weakened inhibitory synaptic transmission onto deep-layer pyramidal neurons, contributing to cognitive variability [22]. Given these findings, we sought to investigate the effects of developmental hypothyroidism on the differentiation and maturation of GABAergic neurons, particularly the PV^+^ interneuron subtype, and clarify the roles and significance of Maf and *Mef2c* in the underlying mechanisms.

In the present study, we employed an integrative experimental approach combining a developmental hypothyroidism mouse model, an HT22 cell line, and primary neuron culture assays to comprehensively investigate the upstream–downstream relationship between Maf and *Mef2c* in neural differentiation of hippocampal PV^+^ interneurons under conditions of developmental hypothyroidism. To model hypothyroidism in vivo, pregnant mice were administered PTU, a well-established pharmacological inducer of hypothyroidism, enabling the investigation of TH function in nervous system development [23,24,25]. Our findings indicate that developmental hypothyroidism can significantly reduce PV^+^ interneurons, which correlates with memory impairment. Mechanistically, this reduction is likely mediated, at least in part, by the downregulation of Maf and its downstream target *Mef2c*. This study could provide a foundation for further investigation of the molecular mechanism by which Maf regulates cognition and memory impairment induced by developmental hypothyroidism, while also identifying potential therapeutic targets for the treatment of this neurodevelopment disorder.

## 2. Materials and Methods

### 2.1. Animals

*C57BL*/*6J* female mice (8 weeks old) were bought from the Experimental Animal Centre of Soochow University (Suzhou, China). All experimental procedures were performed in accordance with the Animal Care and Use Committee at Soochow University. Animal sacrifice was minimized whenever possible. Five *C57BL*/*6J* mice (weighed about 20 g) were housed per cage, on a 12 h light/12 h darkness cycle, at 22 ± 1 °C, and with free access to food and water. Pregnancy was confirmed by detecting vaginal mucus plugs, and the day after the discovery of the vaginal plugs was designed to be embryonic day 1 (E1) and the day after delivery was designed to be postnatal day 1 (P1). Male mouse offspring were utilized in all experiments.

### 2.2. Mouse Model of Developmental Hypothyroidism

To establish a mouse model of developmental hypothyroidism, male and female mice were housed in the same cage in a ratio of 1: 2 for mating. The detection of mouse pregnancy following mating was confirmed by the presence of a vaginal plug early the next morning. Pregnant mice were divided randomly into two groups as follows: (i) pregnant mice in the control group (Con) received tap water only; (ii) in the experimental group, pregnant mice were administrated 50 ppm of 6-Propyl-2-thiouracil (PTU) (Sigma-Aldrich, St Louis, MO, USA, P3755) in drinking water from gestational day 1 to postnatal day 21 (P21), followed by daily replacement of fresh drinking water containing PTU. PTU, an organic compound, potently inhibits thyroid peroxidase and deiodinase in the thyroid gland. This dual inhibition reduces T4 and T3 synthesis while suppressing peripheral T4-to-T3 conversion [26,27].

### 2.3. Assays for Serum Thyroid Hormones

The mice were euthanized with a guillotine. Blood samples (800 μL) were collected from both dams (*n* = 6/group) and offspring pups (*n* = 6/group) at P21 in the control and PTU-treated experimental groups and incubated at room temperature (RT) for 45 min. After blood clotting, the blood sample was centrifuged for 20 min at 4000× *g* and serum was separated. Serum samples were stored at −80 °C for subsequent measurement of free triiodothyronine (FT3), free thyroxine (FT4), and thyroid-stimulating hormone (TSH). After all samples were collected, an enzyme immunoassay was performed to measure the aforementioned hormone levels according to the instructions of the kit manufacturers (Mlbio, Shanghai, China, ML057763, ML301038, ML063200). Each sample and standard were performed in triplicate.

### 2.4. Cell Culture

#### 2.4.1. Primary Hippocampal Neuron Culture

Primary hippocampal neurons were isolated from embryonic brains of mice exposed to PTU and normal mice at 14 days of pregnancy. Hippocampal tissues were dissected out, collected in Hibernate-E media (Thermo, Cambridge, UK, A1247601), and digested using 0.125% Trypsin-EDTA for 25 min at 37 °C. Following a mild trituration, the dissociated cells were seeded onto poly-L-lysine (Sigma-Aldrich, St Louis, MO, USA, P4158)-coated Ø12-mm glass coverslips in 24-well culture plates at a density of 3.5 × 10^5^ cells/cm^2^. Cells were maintained in neurobasal media (supplemented with B27, GlutaMAX, penicillin, and streptomycin) and incubated at 37 °C under 5% CO_2_ and 95% air. Four days later, cells were collected for the following morphological study.

#### 2.4.2. HT22 Cell Line Culture

HT22 cells were cultured in medium (DMEM) with 10% FBS and 1% P/S (Pricella, Wuhan, China, CM-0697) at 37 °C and 5% CO_2_. When 95% confluency was reached, HT22 cells were passaged to 6-well culture plates at 3.5 × 10^6^ cells/cm^2^ for transcriptional and proteomic analysis. After 48 h, total RNA and protein were isolated for quantitative real-time polymerase chain reaction (qRT-PCR) and immunoblot analysis.

### 2.5. Novel Object Recognition (NOR) Test

Sixteen *C57BL*/*6J* adult mice at the age of 14 weeks in the Con (*n* = 8) and PTU group (*n* = 8) were used to perform a NOR test. The NOR test consists of three phases: an adaptation phase, a training phase, and a test phase. Prior to the beginning of the test, the animals received a three-day behavior room environmental adaptation. In brief, mice were habituated to a 10 min exposure session in an open field (40 × 40 × 40 cm) and were free to explore the objects. The next day, the animals received a 10 min training session with two identical objects placed in the open field at regular intervals. The training session was followed by a subsequent 5 min testing on each mouse. Within testing, one of the objects was replaced by a new object and placed in the original position, and the mouse activities were captured using a video camera with an image tracking system (ANY-maze, Wood Dale, IL, USA). The recognition index was calculated as time spent exploring the new object over the total time exploring both objects.

### 2.6. Y-Maze Test

After the NOR test experiment, the mice (*n* = 8 for Con; *n* = 8 for PTU) were continuously used to measure spatial working and reference memory by means of a Y-maze apparatus (arm length: 30 cm; arm bottom width: 5 cm; arm upper width: 10 cm; height of wall: 50 cm). The test involved two separate phases: an adaptation phase and a test phase. In the adaptation phase, one of the arms was designated as a start arm, and the mice were allowed to explore all open arms freely for 5 min. After 2 h, the test phase began and all arms were open. The number of entries into the arms and alternations were recorded for 5 min with an image tracking system (ANY-maze, Wood Dale, IL, USA). Lastly, the percentage of relative alternations was calculated as number of correct alterations/(number of total arms entries-2), as described previously [28].

### 2.7. Dark Avoidance Experiment

The same batch of mice (*n* = 8 for Con, *n* = 8 for PTU) was utilized to carry out a dark avoidance experiment. Notably, the test consists of three phases: an adaptation phase, a training phase, and a test phase. In detail, in the adaptation phase, mice were placed in a light room and allowed to move freely for 5 min through a door hole between the light and dark rooms. In the training phase, the mice were also placed in the light room. When the mice entered the dark room due to the light avoidance response, electrical stimulation was conducted on the mice’s feet and maintained for 10 s. This training allowed the mice to adopt an unconditioned stimulus (electric shocks to the feet) and a conditioned stimulus (dark room) to diminish internal distinctions. In the test phase, the mice were returned to the light compartment. Concomitantly, the time during which the mice moved freely from the bright room into the dark room was recorded as a step-through latency, and the maximum latency was set at 300 s. The times mice entered the dark room were called error times and recorded as well. If the mice did not enter the dark room, the step-through latency was 300 s.

### 2.8. Morris Water Maze (MWM) Test

To evaluate the effects of developmental hypothyroidism-induced impairments on spatial learning and memory, we conducted an MWM test (*n* = 8 for Con; *n* = 8 for PTU) in a circular tank, 120 cm in diameter and 50 cm in height, with water with titanium dioxide at 22 °C. In brief, the circular pool was randomly divided into four equal quadrants (E, east; W, west; S, south; N, north), and a submerged escape platform (10 × 10 cm) was equipped 1 cm below the cloudy water surface in quadrant N. Before the test, the mice had to undergo a 5-day successional adaptive training as described in detail previously [29]. After 24 h, the integrity and capacity of spatial memory was measured according to a previous method [30]. Lastly, the number of platform shuttles and latency to the platform were recorded and tracked by an image tracking system (ANY-maze, Wood Dale, IL, USA).

### 2.9. Immunofluorescence

Animals at two treatment time points (P21 and 14w) was anesthetized with an intraperitoneal injection of chloral hydrate (40 mg/kg) and transcardially perfused with 0.9% cold normal saline followed by 4% paraformaldehyde in 0.1 M phosphate-buffered saline (PBS) (pH 7.4). Subsequently, the brains were dissected, post-fixed in buffered paraformaldehyde overnight at 4 °C, cryoprotected in 30% sucrose in 0.1 M PBS, and sectioned at 12 μm thickness for immunofluorescent staining analysis. Briefly, the sections were rehydrated in PBS for 10 min, followed by incubation with 0.1% Triton X-100 in PBS for 10 min and 5% goat serum in PBS for 1 h at RT. After washing 3 times in PBS for 5 min, the appropriate primary antibodies (DCX, Proteintech, Chicago, IL, USA, 13925-1-AP, 1:200; GAD67, Abcam, Cambridge, UK, ab26116, 1:200; VGLUT2, Sigma, St Louis, MO, USA, MAB5504, 1:200; Parvalbumin, Synaptic Systems, Gottingen, Germany, 195002, 1:200) were incubated at 4 °C overnight. Concomitantly, these sections were incubated with the secondary antibody (Thermo, Cambridge, UK, Alexa Fluor 488, Alexa Fluor 594, 1:500) for 1 h as described in previous studies [31]. For hippocampal primary neurons from E14 brains on glass coverslips, the immunofluorescence procedure was same as the aforementioned. Finally, the sections were mounted onto glass slides for microscopic analysis after being counterstained with DAPI and following full washing with PBS.

### 2.10. Quantitative Polymerase Chain Reaction (Q-PCR)

Total RNA was extracted from fresh hippocampal tissue or HT22 cells (*n* = 6) using RNAiso Plus (TAKARA, Kyoto, Japan, 9108) according to the manufacturer’s instructions. Reverse transcription of 500 ng of total RNA was performed using the RevertAid First Strand cDNA Synthesis Kit (Thermo, Cambridge, UK, K1622). The relative mRNA level was detected by RT-qPCR using a Bio-Rad CFX96 PCR System (Bio-Rad Laboratories, Hercules, CA, USA) and normalized against GAPDH using the 2^−△△CT^ method. The primer sequences for genes of interest were as follows: (1) *Ambra1*; forward primer: 5′-GAAGGTGGCTCTCAGGCATCT; reverse primer: 3′-GCGTCTCAGGTCACATTGAAGC; (2) *Mul*; forward primer: 5′-AAGGAGCTGTGCGGTCTGTT; reverse primer: 3′-CAAAGGTGGGTAGTTCGGTTCC; (3) *Maf*; forward primer: 5′-CCTCTTGAAGCGGCAGGACT; reverse primer: 3′-TCGGACGAGCAGTTGGTGAC; (4) *Mef2c*; forward primer: 5′-AGTCGGCTCAGTCATTGGCTAC; reverse primer: 3′-GCGTGGTGTGTTGTGGGTATC; (5) *Dlx2*; forward primer: 5′-CGCTTCTCCTCCTTGTGCCT; reverse primer: 3′-GGTGGTGATGGTGGTGATGGT; (6) *Wnt5a*; forward primer: 5′-GCGAAGACAGGCATCAAGGAAT; reverse primer: 3′-GCGAAGCGGTAGCCATAGTC. The results were confirmed in at least three separate analyses.

### 2.11. Western Blot

The proteins were extracted from mouse hippocampal tissues with different treatments, homogenized in RIPA lysis buffer (Beyotime Biotech, Shanghai, China, P0013B), and denatured at 95°C for 10 min. Subsequently, a total of 25 μg of each sample was separated by 10% or 12.5% SDS–PAGE and immunoblotted with the following antibodies: polyclonal anti-GAD67 (Abcam, Cambridge, UK, ab26116), polyclonal anti-VGLUT2 (Sigma, St Louis, MO, USA, MAB5504), monoclonal anti-DCX (Zenbio, Chengdu, China, R381606), polyclonal anti-Parvalbumin (Synaptic Systems, Gottingen, Germany, 195002), monoclonal anti-Maf (Zenbio, Chengdu, China, R389104), polyclonal anti-Mef2c (Proteintech, Chicago, USA, 10056-1-AP), monoclonal anti-β-Actin (Proteintech, Chicago, USA, 66009-1-Ig), monoclonal anti-GAPDH (Proteintech, Chicago, USA, 60004-1-Ig), and monoclonal anti-β-Tubulin (Zenbio, Chengdu, China, R380992). All primary antibody dilutions were applied according to the manufacturer’s instructions. Notably, β-Actin, GAPDH, or β-Tubulin was used as an internal control. After repeated washing with PBS, the immunoblots were visualized using enhanced chemiluminescence and captured using a gel systems camera. 

### 2.12. siRNA Knockdown and Plasmid Transfection

HT22 cells were seeded in six-well plates. When the cells reached 70% confluency, 75 nM siRNA was delivered into the cultures of HT22 cells using a GP-transfect-Mate transfection reagent (GenePharma, Shanghai, China), and the cells maintained for 48 h after the initial transfection. Subsequently, the cells were harvested for the next experiment. Noteworthily, in knockdown tests, RNA with no homology to the target gene was employed as the control siRNA. In overexpression experiments, plasmids containing the *Maf* gene were also synthesized by GenePharma, and 2.5 μg plasmids was applied for the transfection of HT22 cells by means of lipofectamine 3000 (Thermo, Cambridge, UK, L3000075) according to the manufacturer’s instructions.

### 2.13. Quantification and Statistical Analysis

Statistical analyses were performed using Graph Pad Prism 8.0. All statistical tests were performed using Student’s *t*-test. All results are expressed as mean ± SEM from at least three independent experiments. *p* < 0.05 was considered statistically significant.

## 3. Results

### 3.1. Generation and Characteristics of Developmental Hypothyroidism and Identification of Serum Hormone (FT4, FT3 and TSH) Levels in Offspring

**Figure 1:**
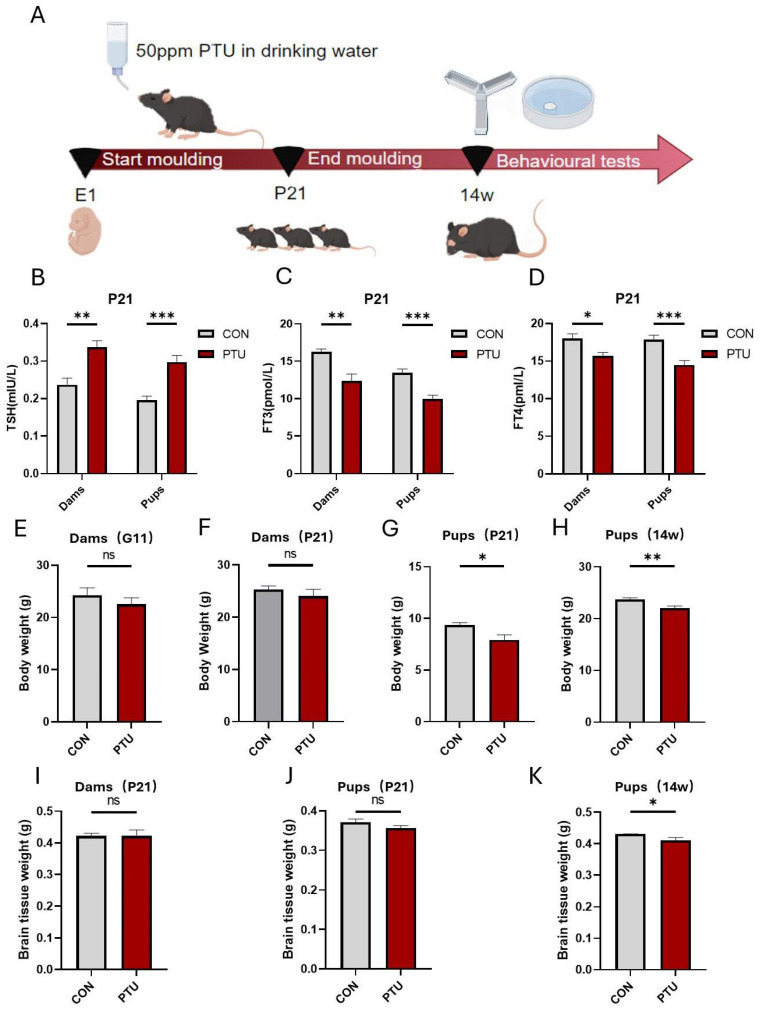
The establishment of an experimental model of developmental hypothyroidism and the identification of thyroid hormone levels. (**A**) Schematic illustrating the establishment of the experimental model of developmental hypothyroidism. Levels of TSH (**B**) and thyroid hormones FT3 (**C**) and FT4 (**D**) in the blood of postpartum dams at 21 days and pups at P21 following developmental exposure to PTU. Changes in body weight in pregnant mice at G11 (**E**) and postpartum dams at 21 days (**F**) following PTU exposure. Body weight of pups at P21 (**G**) and 14w (**H**) after developmental hypothyroidism. (**I**) Brain tissue weight of postpartum dams at 21 days. (**J**,**K**) Brain tissue weight of pups at P21 and 14w, respectively. *n* = 6. CON, control; PTU, developmental exposure to PTU; TSH, thyroid-stimulating hormone; FT4, free thyroxine; FT3, free triiodothyronine; P, postnatal day. Data are represented as mean ± SEM. ns: non-significant, * *p* < 0.05, ** *p* < 0.01, and *** *p* < 0.001 compared with the corresponding controls.

To systematically validate the effects of developmental hypothyroidism on the impairments of cognitive and memory function in offspring and elucidate the underlying mechanisms, we first established a model of developmental hypothyroidism and identified serum hormone (FT4, FT3, and TSH) levels in offspring. In Figure 1A, a schematic illustration depicts the procedure for the generation of developmental hypothyroidism by administrating 50 ppm PTU in drinking water from E1 onwards until P21. Next, we examined the serum TSH levels of the dams and pups. The results showed that treatment with PTU significantly increased serum TSH concentrations (Figure 1B). Contrary to the change in TSH, ELISA analysis demonstrated that the levels of serum THs (FT3 and FT4) of the dams and pups significantly decreased at P21 (Figure 1C,D). The results suggest that the experimental animal model of hypothyroidism was successfully established. Nonetheless, no differences in body weight were found between the pregnant mouse or dam groups during the gestational (G11) and postpartum (P21) stages (Figure 1E,F), while the body weight of pups in the hypothyroid group at P21 and 14w was diminished compared to the controls (Figure 1G,H). In addition, no differences in the weight of brain tissue were found in postnatal dams, or in offspring, at P21 (Figure 1I,J). Strikingly, the difference between the PTU and Con pup groups became larger with the prolongation of time, and a significant difference in brain tissue weight was found at 14w (Figure 1K), suggesting that thyroid hormone deficiency during pregnancy and lactation resulted in postnatal growth retardation in mice.

### 3.2. Effect of Developmental Hypothyroidism on Cognitive and Memory Function in Offspring Mice

**Figure 2:**
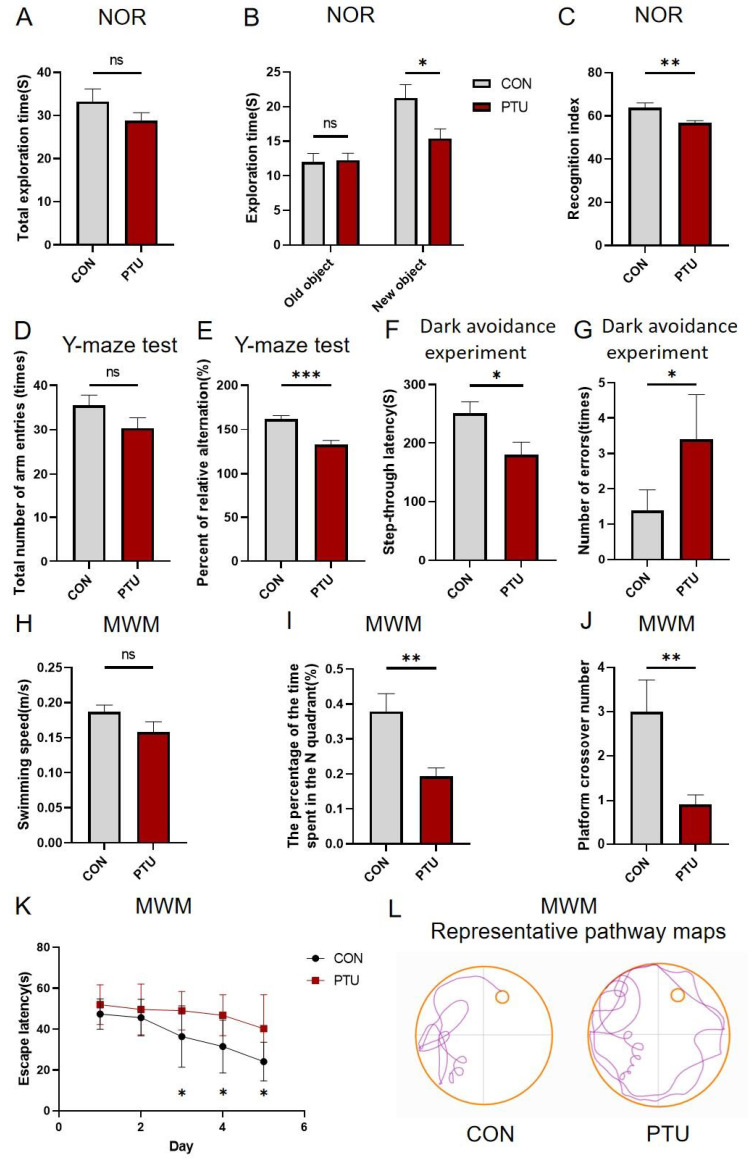
Cognitive and memory alterations in adult offspring mice with developmental hypothyroidism. The assessment of the novel object recognition (NOR) test in mice with developmental hypothyroidism, including total exploration time (**A**), exploration time for new and old objects (**B**), and the recognition index (**C**), were recorded. Note that the recognition index was lower in the PTU group than in CON. The total number of arm entry measurements (**D**) and the percent of relative alternations (**E**) in the Y-Maze. The test of step-through latency (**F**) and number of errors (**G**) in dark avoidance. The Morris Water Maze (MWM) test is associated with swimming speed (**H**), the percentage of time spent in the N quadrant (**I**), platform crossover number (**J**), escape latency (**K**), and representative pathway maps (**L**). Note that memory was impaired in developmental hypothyroidism mice exposed to PTU at 14w. CON, control; PTU, developmental exposure to PTU. *n* = 8; all data are reported as means ± SEM. ns: non-significant, * *p* < 0.05, ** *p* < 0.01, *** *p* < 0.001 compared with the corresponding controls.

To reveal whether there was cognitive and memory impairment in offspring following maternal hypothyroidism during pregnancy and lactation, spatial learning and memory were assessed by the novel object recognition test (NOR), Y-Maze test, dark avoidance experiment, and Morris Water Maze test (MWM). As shown in Figure 2, the cognition and memory of offspring mice born to dams exposed to 50 ppm PTU during pregnancy and lactation were impaired when compared to normal mice. In NOR, the PTU offspring mice showed no significant difference in total exploration time and exploration time of the old object (Figure 2A,B) but a significantly reduced exploration time of the new object and recognition index were detected compared to the offspring mice from the Con group (Figure 2B,C). In the Y-Maze test, the PTU offspring mice showed same total number of arm entries as the Con offspring mice (Figure 2D), but significantly fewer spontaneous alternating behaviors were found (Figure 2E). Consistently, in the dark avoidance experiment, there was a remarkable decrease in spatial learning and memory in PTU-induced animals compared to controls, as evidenced by shorter latency (Figure 2F) and increased error frequency (Figure 2G). Likewise, PTU and Con offspring mice showed no difference in swimming speed (Figure 2H). However, PTU offspring mice spent less time in the platform quadrant (N) (Figure 2I), had fewer platform shuttles (Figure 2J), and needed more time to find the platform (Figure 2K,L). Thus, these data implicate a learning memory impairment in offspring caused by TH-deficient prenatal and lactation stages.

### 3.3. Influence of Developmental Hypothyroidism on Generation of Neuronal Precursor Cells in Offspring Hippocampus

**Figure 3:**
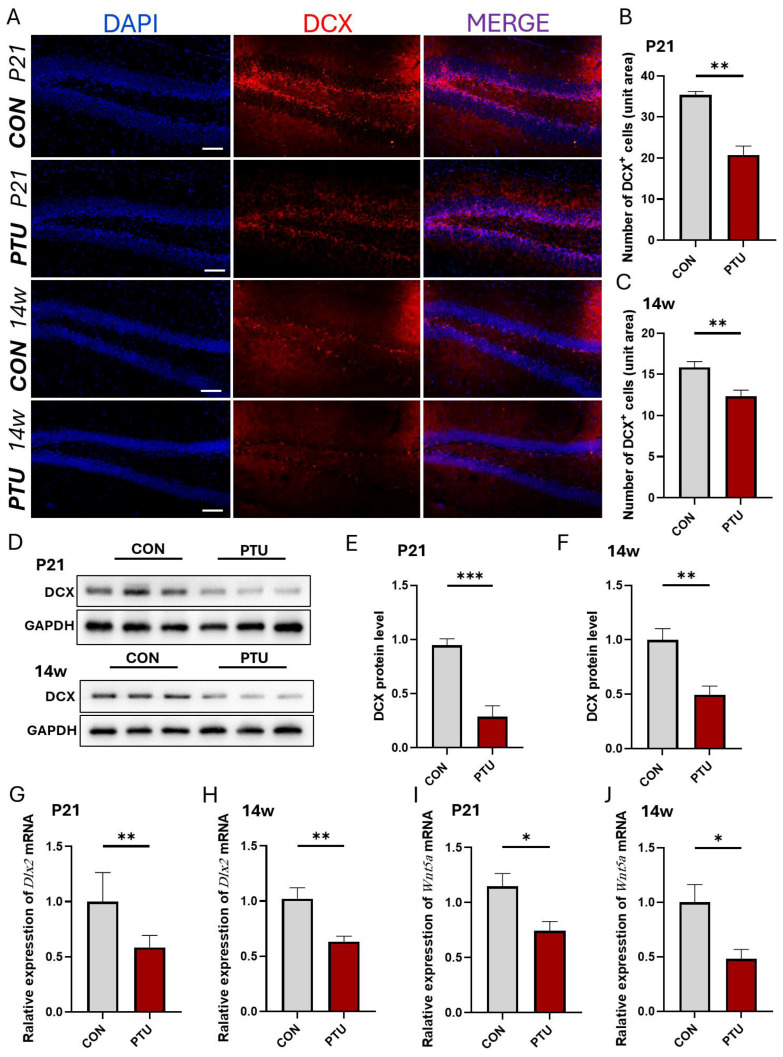
Changes in the generation of neuronal precursor cells in the juvenile and adult hippocampus after developmental hypothyroidism. (**A**) Immunofluorescence of DCX (red) in the subgranular region of the dentate gyrus of the hippocampus in juvenile (P21) and adult (14w) mice with developmental hypothyroidism. (**B**,**C**) Quantitative analysis of DCX-positive neurons under the indicated treatment at P21 and 14w, respectively. (**D**) Representative Western blot bands showing levels of DCX in the hippocampus of mice undergoing the indicated treatment at P21 and 14w. (**E**,**F**) Quantitative analysis of the levels of DCX at P21 and 14w normalized to GAPDH, respectively. (**G**,**H**) Real-time PCR showing the mRNA expression levels of the differentiation-associated gene *Dlx2* in the hippocampus of mice undergoing the indicated treatment at P21 and 14w, respectively. (**I**,**J**) The mRNA level of *Wnt5a* in the hippocampus of mice undergoing the indicated treatment at P21 and 14w. Data are reported as means ± SEM of three independent experiments. Scale bars = 100 μm. * *p* < 0.05, ** *p* < 0.01, *** *p* < 0.001 vs. their respective controls.

To reveal whether neurogenesis and neuronal development are affected following developmental hypothyroidism in offspring mice, we assessed the number of neuronal precursor cells positive for doublecortin (DCX), a marker for neuronal precursors and neurogenesis, in the hippocampal dentate gyrus, which is intimately associated with spatial learning and memory. As shown in Figure 3A–C, DCX-positive cells significantly decreased in the PTU group at the age of P21 and 14w in offspring. Similar to quantitative DCX^+^ cells, Western blot analysis further showed that the protein level of DCX in the Con group displayed higher expression and had less double expression in the PTU group, especially at P21 and 14w (Figure 3D–F). To further validate this, we detected the expression of Distalless-related homeobox (*Dlx*), a special molecule responsible for neurogenesis, proliferation, and differentiation in the nervous system, using Q-PCR. Our result showed that the relative expression of mRNA in the hippocampus from the PTU group was significantly reduced at P21 and 14w compared to Con group (Figure 3G,H). Intriguingly, the relative expression of *Wnt5a*, which is involved in regulating proliferation, commitment, and differentiation in neuronal progenitor cells during development, also showed lower expression levels at P21 and 14w (Figure 3I,J). These data suggest that developmental hypothyroidism leads to neuronal proliferation and differentiation abnormalities, including a reduction in neuronal precursor cells in juvenile mice until adulthood.

### 3.4. Preferential Changes in Hippocampal GABAergic Interneurons in Offspring of Hypothyroid Mice

**Figure 4:**
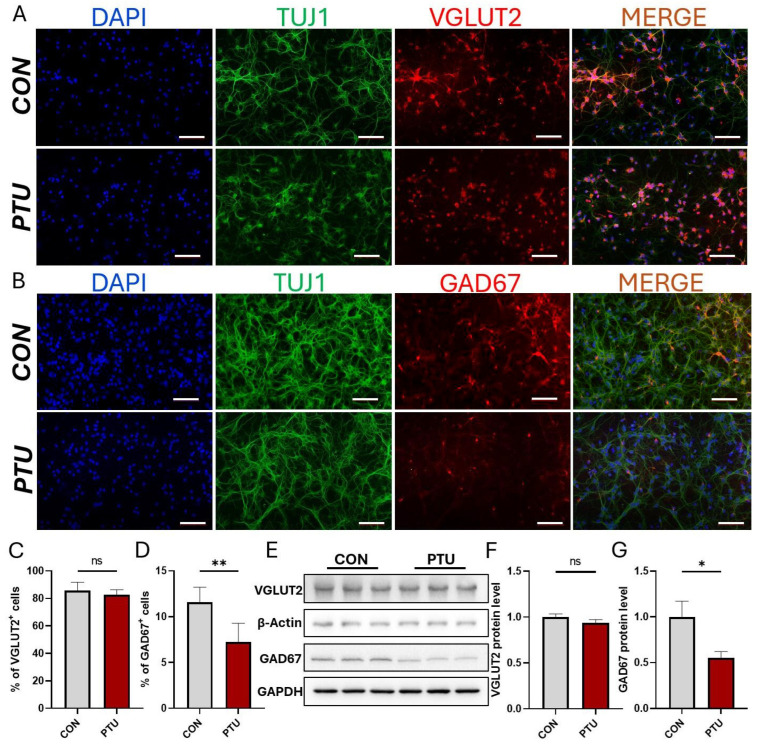
Effects of developmental hypothyroidism on hippocampal glutamatergic and GABAergic neuronal differentiation in vitro. (**A**) Immunofluorescence revealed expression of glutamatergic neuron marker VGLUT2 in primary hippocampal cells from CON and PTU mice. (**B**) Immunostaining for GABAergic neuron marker GAD67 in primary hippocampal cells of CON and PTU mice. (**C**,**D**) Quantitative assessment of the percentage of VGLUT2^+^ and GAD67^+^ cells in the CON and PTU groups, respectively. (**E**) Western blot analysis of VGLUT2 and GAD67 expression in primary hippocampal cells following developmental hypothyroidism. (**F**,**G**) Quantification of expression levels of VGLUT2 and GAD67 normalized to β-actin. *n* = 6. Data are presented as mean ± SEM of three independent experiments. Scale bars = 100 μm. ns: non-significant, * *p* < 0.05; ** *p* < 0.01.

To further determine which type of neuronal development in offspring is affected by developmental hypothyroidism, primary neurons were prepared from each group. Five days later, VGLUT2- and GAD67-positive cells were evaluated by parallel immunofluorescence analysis. As shown in Figure 4A,C, the number of VGLUT2-positive neurons showed no difference between the PTU and Con groups. Surprisingly, the number of GAD67-positive neurons significantly decreased in the PTU group compared with the Con group (Figure 4B,D). In line with this result, Western blot analysis for VGLUT2 expression in primary hippocampal neurons also showed no significant changes (Figure 4E,F). In contrast, Western blot analysis also revealed a significant decrease in the expression of the GAD67 protein in primary hippocampal neurons (Figure 4E,G).

**Figure 5:**
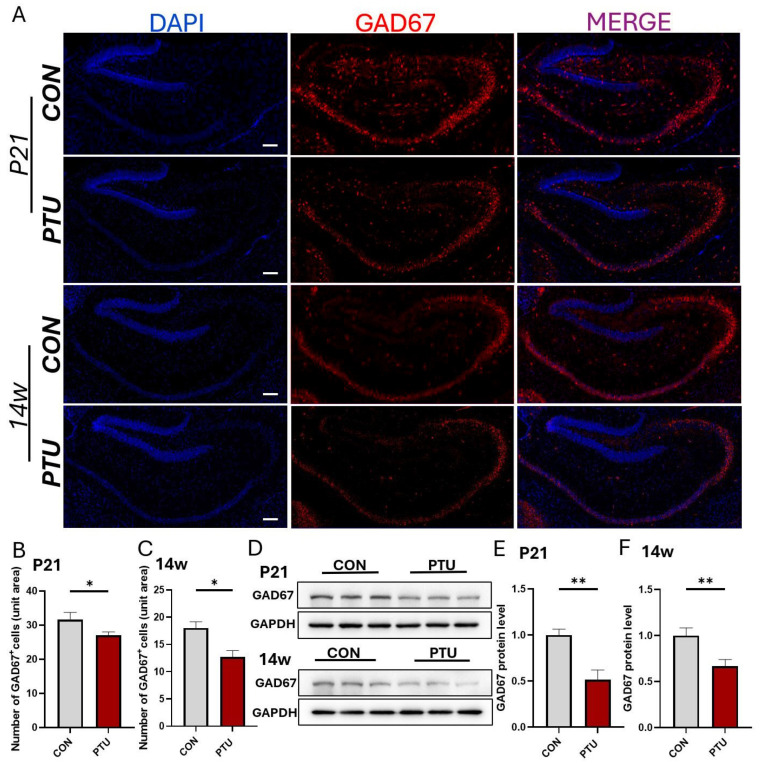
Changes in glutamatergic and GABAergic neurogenesis in adolescent and adult mouse hippocampal region following developmental hypothyroidism. (**A**) Representative images of immunostaining of GAD67 (red) in male mice at P21 and 14w following developmental hypothyroidism. (**B**,**C**) Quantification of GAD67-positive neurons in mice hippocampus at the ages of P21 and 14w, respectively. (**D**) Western blot analysis of GAD67 expression in mouse hippocampus at the ages of P21 and 14w following the developmental hypothyroidism, respectively. (**E**,**F**) Quantification of expression levels of GAD67 normalized to β-actin at the ages of P21 and 14w. Note that a similar reduction tendency in GAD67 protein expression was observed at P21 and 14w. *n* = 6. All data are presented as mean ± SEM. Scale bars: 200 μm. * *p* < 0.05; ** *p* < 0.01.

In addition, we assessed the changes in the expression of GAD67 and VGLUT2 protein levels and the number of GABAergic neurons and glutamatergic neurons in the hippocampal dentate gyrus. As shown in Figure 5A–C, the expression of GAD67 levels and the number of GAD67-positive neurons in the hippocampus were significantly reduced in the PTU group at P21 and 14w compared with the corresponding Con groups, respectively. Consistent with the immunofluorescence results, Western blot analysis revealed similar expression patterns of GAD67; namely, there was a significant decrease in the expression of the GAD67 protein in the hippocampus at both P21 and 14w (Figure 5D–F). As for the expression of VGLUT2 protein levels and the number of glutamatergic neurons, no data are shown because no significant changes were found between two groups at the same two aforementioned stages.

### 3.5. Change in PV^+^ Neurons, a Subtype of GABA Interneurons, in Hippocampus of Hypothyroid Mouse Offspring

**Figure 6:**
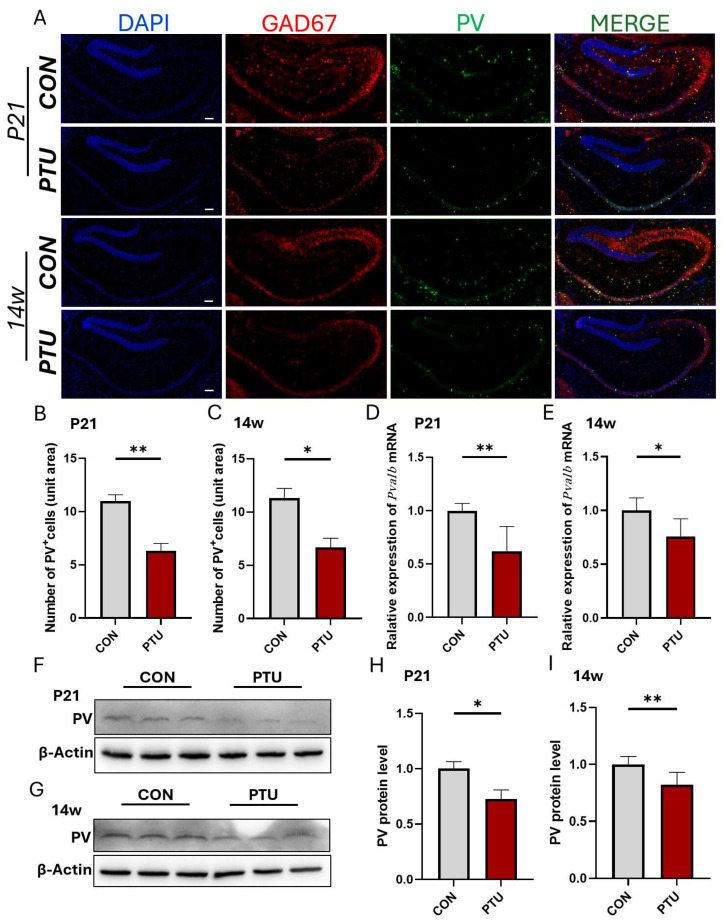
Changes in the neurogenesis of PV^+^ interneurons as a subtype of GABAergic neurons in juvenile and adult mouse hippocampus following developmental hypothyroidism. (**A**) Representative images of immunofluorescent double staining for GAD67 (red) and PV (green) in mouse hippocampus at P21 and 14w. Note that PV^+^ interneuron is the most abundant GABAergic neuron subtype. PV, parvalbumin. (**B**,**C**) Quantification of the number of PV-positive neurons. (**D**,**E**) Q-PCR revealed the changes in *Pvalb* at the transcriptional level in the PTU group compared to the CON group at P21 and 14w. (**F**,**G**) Western blot analysis of parvalbumin expression in the hippocampus under the indicated treatment. (**H**,**I**) Quantification of expression levels of parvalbumin normalized to β-actin. Data are represented as mean ± SEM of three independent experiments. Scale bar = 200 μm (**A**). * *p* < 0.05; ** *p* < 0.01 vs. their respective controls.

Next, we detected the changes in PV^+^ neurons, the most abundant subtype of GABA interneurons, in the hippocampus of hypothyroid mouse offspring. As shown in Figure 6A–C, PV^+^ interneurons were significantly reduced at P21 and 14w in the PTU group as compared with the Con group. Moreover, Q-PCR analysis showed that the mRNA levels of Pvalb, a specific marker for PV^+^ interneurons, were drastically downregulated at P21 and continuously reduced until 14w (Figure 6D,E). In line with Q-PCR results, Western blot assay further validated that the expression of parvalbumin protein in the hippocampus similarly declined at P21 and 14w (Figure 6F–I).

### 3.6. Possible Mechanism Underlying Cognitive and Memory Impairment in Offspring Arising from Abnormal Development of PV^+^ Interneurons

**Figure 7:**
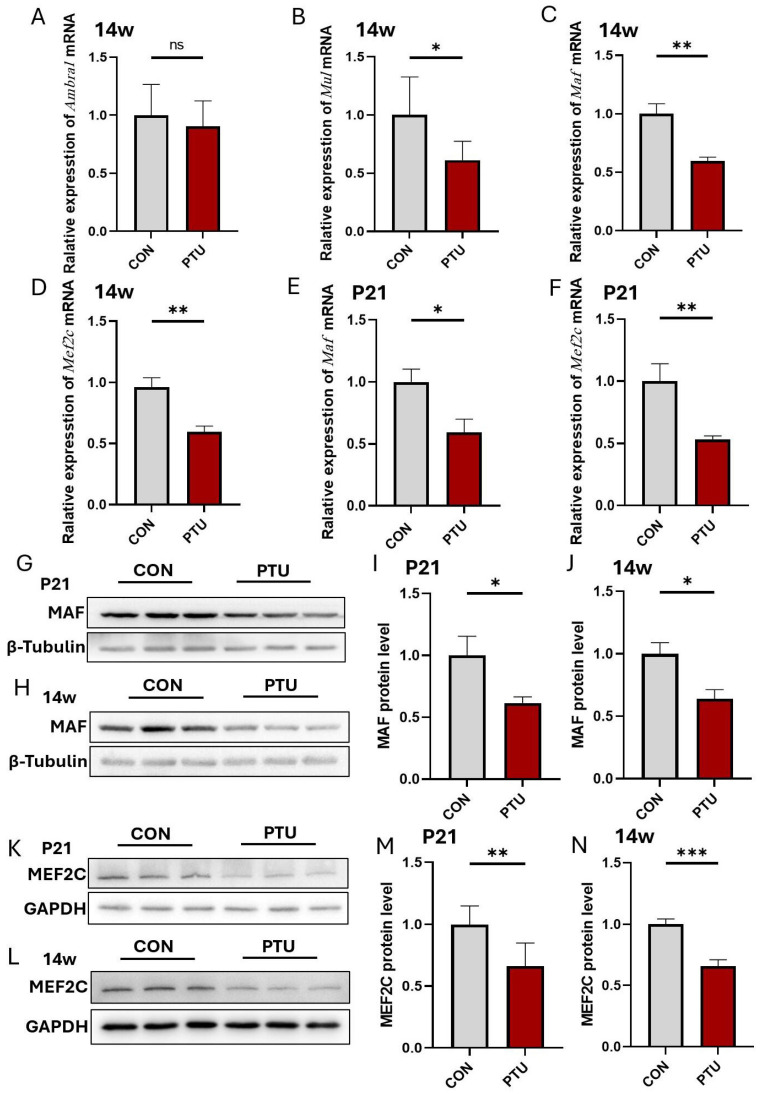
Effects of developmental hypothyroidism on the expression of genes related to hippocampal PV^+^ neuronal differentiation in mouse offspring. (**A**–**D**) mRNA expression for hippocampal PV^+^ neuronal differentiation-related genes *Ambra1* (**A**), *Mul* (**B**), *Maf* (**C**), and *Mef2c* (**D**) in adult (14w) CON and PTU mouse offspring using real-time RT-PCR. Furthermore, mRNA for *Maf* (**E**) and *Mef2c* (**F**) in juvenile (P21) CON and PTU mouse offspring was also assayed. (**G**–**J**) Western blot and quantitative analysis of Maf in juvenile (P21) and adult (14w) mouse hippocampus. (**K**–**N**) Western blot and quantitative analysis of Mef2c in juvenile (P21) and adult (14w) mouse hippocampus. *n* = 6. Data are reported as means ± SEM of three independent experiments. ns: non-significant, * *p* < 0.05, ** *p* < 0.01, and *** *p* < 0.001 compared with the corresponding controls.

To further investigate the molecular mechanisms underlying the reduced PV^+^ interneurons resulting in cognitive and memory impairment in offspring, several genes associated with PV^+^ interneurons were identified through certain-scale screening of hippocampal tissues. The results showed no significant difference in the expression of the *Ambra1* gene in the PTU group compared to the Con group at 14w (Figure 7A), but the *Mul*, *Maf*, and *Mef2c* genes appeared significantly downregulated at 14w in developmental hypothyroidism mice, especially the *Maf* and *Mef2c* genes (Figure 7B–D). Similarly, Q-PCR analysis also showed a significant downregulation of *Maf* and *Mef2c* mRNA in P21 mice with developmental hypothyroidism (Figure 7E,F). Western blot further validated the downregulation of Maf (Figure 7G–J) and Mef2c (Figure 7K–N) in developmental hypothyroidism mouse pups at P21 and 14w.

**Figure 8:**
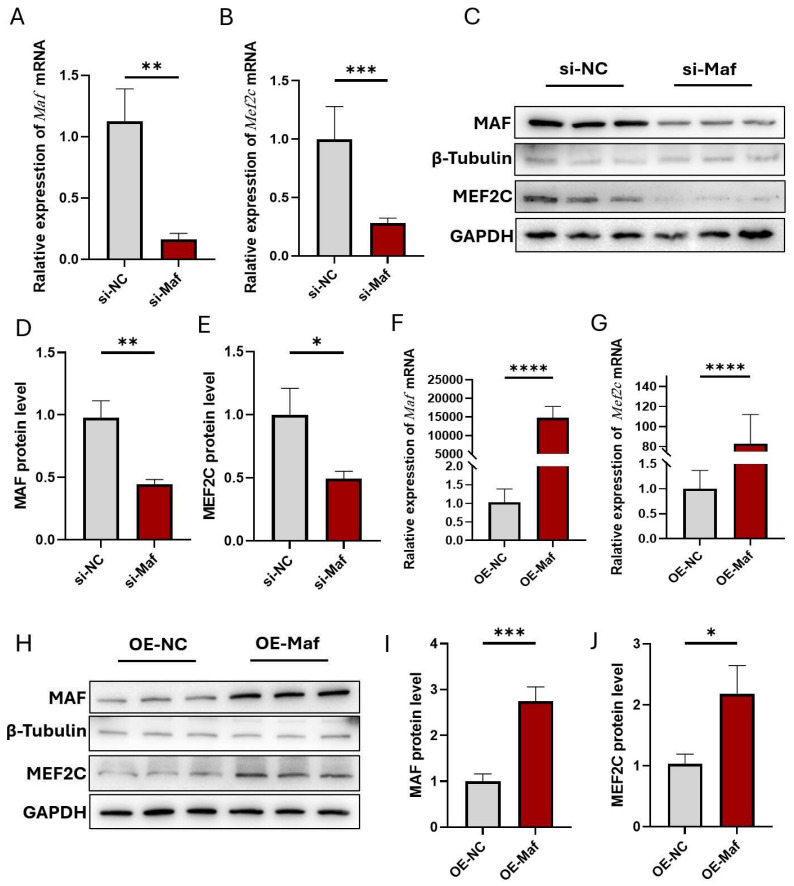
The regulatory effect of Maf on its downstream signaling molecule *Mef2c* related to hippocampal PV^+^ neuronal differentiation. (**A**) mRNA expression of the *Maf* gene in HT22 cells following transfection with siRNA using Q-PCR. (**B**) *Maf* knockdown by siRNA results in a decrease in the mRNA expression of *Mef2c* in HT22 cells. (**C**) Western blot analysis of Maf and Mef2c expression under the indicated conditions. (**D**,**E**) Quantification of expression levels of Maf and Mef2c normalized to GAPDH. (**F**) Examination of overexpression of *Maf* in HT22 cells using Q-PCR. (**G**) Q-PCR was used to detect the effect of *Maf* overexpression on *Mef2c* expression in HT22 cells. (**H**) Western blot analysis of transfection of the *Maf* gene and Mef2c protein expression in HT22 cells. (**I**,**J**) Quantification of the levels of *Maf* overexpression and Mef2c expression in the indicated condition, respectively. si-NC, si-negative control. *n* = 6. All data are reported as means ± SEM. * *p* < 0.05, ** *p* < 0.01, *** *p* < 0.0001, and **** *p* < 0.0001 vs. their respective controls.

Given that Maf and Mafb regulate mouse interneuron fate specification and maturation, and Maf is likely to contribute to the regulation of *Mef2c* expression, we further investigated whether Maf can regulate *Mef2c* expression levels to strengthen the differentiation toward PV^+^ interneurons. As shown in Figure 8A, the mRNA level of *Maf* was significantly reduced when HT22 cells were transfected with *Maf* siRNA. Notably, *Maf* knockdown substantially decreased the mRNA level of *Mef2c* (Figure 8B). In addition, *Maf* knockdown caused a significant downregulation of Mef2c protein (Figure 8C–E). Conversely, an overexpression of *Maf* could lead to a marked increase in the expression of *Mef2c* (Figure 8F–J). These results imply that Maf is involved in the regulation of *Mef2c* expression and affects the determination of PV^+^ interneurons’ fate during development.

## 4. Discussion

THs play an indispensable role in the growth and development of various tissues. In addition, THs also regulate several metabolic processes in both the mother and the embryos during pregnancy and lactation. Given that fetal thyroid function is still inactive as early as E11.5–E12.5, placental and fetal development still depends on maternal THs in early gestation [32]. Although the fetal thyroid gland is functionally mature in late pregnancy, the availability of maternal THs to fetal development is still considerable. Until now, accumulating studies have shown that THs are also important for brain development throughout the fetal and postnatal stages [33]. Strikingly, loss or deficiency of THs during pregnancy in different species leads to behavioral and memory deficits in the offspring of affected animals. Although an increasing number of studies have revealed that developmental hypothyroidism impairs brain functions, including IQ, emotional regulation, and behavioral skills, in juvenile and adult offspring, little is known about the specific neuronal damage and molecular mechanisms of developmental hypothyroidism-induced memory deficiency.

In this study, we present in vitro and in vivo data elucidating the effects of TH deficiency on hippocampus-specific neuronal development and, as a result of it, on cognitive and memory impairments, as well as the upstream–downstream relevance between Maf and *Mef2c* in the neural differentiation of hippocampal PV^+^ interneurons in developmentally hypothyroid mice. Our results convincingly demonstrated that developmental hypothyroidism significantly resulted in cognitive and memory impairments and neurogenesis and differentiation abnormalities, especially in hippocampal GABAergic interneurons. More importantly, TH deficiency-induced PV^+^ interneuron reduction could substantially contribute to cognitive and memory impairment in offspring via Maf-mediated downregulation of *Mef2c* expression. Therefore, the present study illustrating developmental hypothyroidism-induced cognition and memory impairment could provide an important insight into a potential therapeutic target for the prevention and treatment of this neurodevelopmental disorder.

It was previously reported that TH deficiency in mice during gestation and lactation causes hippocampal abnormalities and spatial learning memory impairments in offspring [34]. In the present study, we also found that the model of developmentally hypothyroid mice via administration of PTU showed functional impairments in learning and memory behaviors in juvenile and adult male offspring but without a striking impact on motor ability. These results reveal the crucial roles of THs in maintaining the normal function of the nervous system and further validate the hippocampal memory deficiency arising from developmental hypothyroidism. This behavior status is in line with previous reports. Nevertheless, it is quite necessary to quickly clarify the underlying mechanisms to find a precise therapeutic target to counteract the harm.

It is well-known that defects of the neurological functional structure, such as hippocampal neurodevelopmental abnormality following developmental hypothyroidism, contribute to memory deficits in adult animals [5,35]. However, the causal relationships between neuronal development and memory impairment have still not been established. Additionally, it is largely unknown whether neural precursor cells associated with learning and memory behaviors are particularly affected during differentiation and development. Based on these points, we further investigated the differentiation of neuronal precursor cells in special regions following developmental hypothyroidism. As we expected, the results show that the capacity of neural precursor cells to give rise to specific neurons significantly declined, displaying a marked decrease in the number of both DCX^+^ cells and newly generated neurons. Therefore, the decline in the number of neuronal cells is likely to be insufficient to persist in intact hippocampus-dependent cognitive and memory ability. Our findings provide strong evidence for the relationship between persistent memory impairment and neurogenesis following developmental hypothyroidism.

The hippocampus is one of the most prominent structures involved in learning, spatial recognition memory, and emotion and information processing, and the principal cell types of neurons in the hippocampus are glutamatergic excitatory and GABAergic inhibitory neurons. Notably, GABAergic neurons demonstrate heightened vulnerability to damage from diverse etiological factors [36,37,38]. The tremendous diversity in interareal axon projection patterns forms the structural basis of the hierarchical network [7,8]. In the hippocampus, PV^+^ interneurons account for 40% of GABAergic interneurons and represent the most common GABAergic interneuron subtype [9,16]. Importantly, PV^+^ interneurons play a critical and instructive role in coordinating CA1 network communication and hippocampal–neocortical dialogue, which drives both long-term network plasticity and memory formation [39,40]. Based on the literature on GABAergic interneurons, we examined the changes in GABAergic interneurons in the hippocampus, especially PV^+^ interneurons, after developmental hypothyroidism. Our results showed that developmental hypothyroidism can impair hippocampal GABAergic interneurons, exhibiting a marked decrease in PV^+^ interneurons, suggesting that developmental hypothyroidism-induced deficiency in learning and spatial recognition memory is likely attributed to the loss or degeneration of PV^+^ interneurons. Meanwhile, our findings also provide a plausible interpretation of the robust and persistent memory impairment arising from developmental hypothyroidism.

Despite the aforementioned data implying an involvement of PV^+^ interneuron loss or degeneration in robust and persistent memory impairment arising from developmental hypothyroidism, the key molecular determinants governing PV+ interneuron differentiation and maturation remain elusive. The topics relevant to persistent memory impairment prompt us to investigate it further. Many previous studies have revealed that Maf and *Mef2c* play a pivotal role in controlling morphological maturation of cortical interneurons and enhancing the generation of PV^+^ interneurons [13]. Similarly, we found that Maf and *Mef2c* were markedly downregulated in mice with developmental hypothyroidism, indicating the involvement of Maf and *Mef2c* in inducing a loss of PV^+^ interneurons. To substantiate this view, we further utilized siRNA and overexpression assays to identify Maf as an upstream regulator of *Mef2c* and to identify the correlation between the two molecules. Our results revealed that *Maf* knockdown caused a significant downregulation of Mef2c at the gene and protein levels. In contrast, overexpression of *Maf* can cause a marked increase in the expression of *Mef2c.* The results also imply that Maf exerts an important role in regulating *Mef2c*, a critical factor for PV^+^ interneuron neurogenesis in the context of the medial ganglionic eminence differentiation system. Maf, a bZIP transcription factor, contains a highly conserved extended homology region or an ancillary DNA binding region, in addition to a typical basic region, and both regions are involved in target DNA sequence recognition [41]. Existing studies have revealed a binding region of Maf in the *Mef2c* gene sequence. Therefore, we speculated that the molecular mechanisms underlying the interaction between Maf and *Mef2c* orchestrate the development of PV^+^ interneurons and develop strategies rendering PV^+^ neurons more resistant to developmental hypothyroidism. Given that juvenile and adult mice with hypothyroidism during pregnancy and lactation acquired long-lasting memory deficits, a decreased interneuron differentiation of neural precursor cells is tightly linked to long-lasting hippocampus-dependent memory deficits following developmental hypothyroidism in male mice. Due to the unique functional properties of PV^+^ interneurons, this allows them to precisely control local circuitry and memory processing. Nevertheless, there is still a limitation in the present study regarding long-lasting cognitive and memory impairment due to reduced hippocampal interneuron differentiation of neural precursor cells following developmental hypothyroidism. There are other mechanisms that involve neuronal apoptosis, and non-neuronal cell dysfunction causing long-lasting cognitive and memory impairment needs to be further addressed. In addition, in the absence of rescue experiments, the observed correlation between Maf-*Mef2c* transcriptional alterations and behavioral phenotypes requires substantiation through additional experimental evidence.

## 5. Conclusions

**Figure 9:**
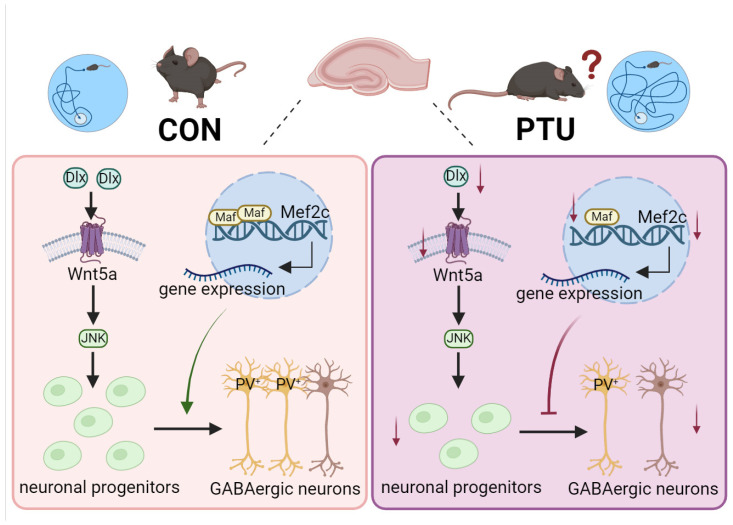
Schematic diagram illustrating the underlying molecular mechanism of memory impairment by which developmental hypothyroidism elicits an aberrant development of PV interneurons, a subtype of GABAergic neurons, by reducing *Mef2c* expression, mainly via the downregulation of Maf. The red arrows indicate the downregulation or reduction.

In summary, our present work identifies that the hippocampus, one of the principal brain regions associated with learning and memory, undergoes cytoarchitecture and functional changes following developmental hypothyroidism in juvenile and adult mice, and this effect is likely due to a reduced differentiation of GABAergic neurons in the hippocampus, especially the PV^+^ interneuron subtype, a subpopulation susceptible to developmental hypothyroidism in juvenile and adult mice. In addition, we also unravel the possible molecular mechanisms responsible for reduced PV^+^ interneurons after developmental hypothyroidism; namely, the reduction is mainly attributed to the downregulation of *Mef2c* modulated by Maf (Figure 9). This study is likely to provide a novel therapeutic targeting strategy for the treatment and prevention of memory disorders.

## Data Availability

The data that support the findings of this study are available from the corresponding author upon reasonable request.
